# Tick Species Identification and Zoonotic Bacteria Detection from Healthcare-Extracted Specimens from Humans in the Basque Country, Northern Iberian Peninsula

**DOI:** 10.3390/pathogens14060561

**Published:** 2025-06-04

**Authors:** Patirke Ibarrondo-Mendiola, Patricia Vázquez, Miriam Alkorta, Cristina Zugazaga, Ana L. García-Pérez, Jesús F. Barandika, Aitor Cevidanes

**Affiliations:** 1 Department of Animal Health, NEIKER—Basque Institute for Agricultural Research and Development, Basque Research and Technology Alliance (BRTA), 48160 Derio, Spain; 2 Department of Microbiology, Donostia University Hospital, 20014 Donostia-San Sebastián, Spain; 3 Department of Microbiology, Basurto University Hospital, 48013 Bilbao, Spain; 4 BioCruces Health Research Institute, 48903 Barakaldo, Spain

**Keywords:** *Ixodes ricinus*, passive entomologic surveillance, human-biting ticks, Lyme disease, zoonotic tick-borne bacteria

## Abstract

Hard ticks are vectors of a wide range of pathogens, with tick-borne bacteria being among the most frequently detected. This study presents a first approach to the identification of human-biting ticks in the Basque Country (northern Iberian Peninsula), where previous research has mainly focused on ticks from vegetation and domestic and wild animals. The aim of this study was (i) to identify tick species collected in hospitals and health centres and (ii) to determine the presence and identify of pathogenic bacteria that they may carry using PCR, RLB and sequencing methods. A total of 181 ticks were collected and grouped in pools of one, two or three specimens, resulting in 157 samples. Morphological and molecular identification of collected ticks revealed that most specimens belonged to *Ixodes ricinus* (88.5%). Additionally, *Rhipicephalus bursa*, *Haemaphysalis punctata*, *Dermacentor reticulatus* and *Dermacentor marginatus* were also identified. A total of 25 samples (15.9%) tested positive for DNA from the targeted pathogens. The most prevalent vector-borne bacteria were *Borrelia* spp. (6.4%), followed by *Rickettsia* spp. (5.1%), *Anaplasma phagocytophilum* (2.5%) and *Coxiella* spp. (1.9%). Species identified included *B. afzelii*, *B. garinii*, *B. miyamotoi*, *B. valaisiana*, *B. burgdorferi* s. s., *R. monacensis*, “*Candidatus* R. rioja”, *R. helvetica* and *R. massiliae*. This study highlights the usefulness of combining molecular diagnostics with passive surveillance of human-attached ticks as an effective tool for regional monitoring of ticks and tick-borne pathogens.

## 1. Introduction

Hard ticks are obligate blood-feeding arthropods that parasitize mammals, birds, reptiles and even amphibians, distributed in almost all regions of the world [1]. Ticks are significant vectors responsible for transmitting a diverse array of pathogens. While viruses and bacteria are the most frequently detected tick-borne agents, protozoa and helminths can also be spread through tick bites [2]. The prevalence of tick-borne diseases (TBDs) caused by tick-borne pathogens (TBPs) depends largely on both the geographic distribution and the activity of their tick vectors [3]. The population dynamics of tick species depend on the effects of biotic and abiotic factors, including climate, vegetation type and host density [4]. Environmental conditions in the Basque Country, a region of the northern Iberian Peninsula with a temperate climate and abundant rainfall throughout the year, favour tick survival and reproduction [5] and 12 species of ticks collected from vegetation have been identified [5]. Moreover, outdoor activities such as forestry work, hunting, mushroom foraging and hiking are commonly practiced in the Basque Country, further increasing the likelihood of human exposure to ticks.

The maintenance of tick-borne bacteria in nature relies on complex transmission cycles involving ticks and a wide range of wild and domestic animal hosts, with humans acting as incidental hosts [3]. Among these bacteria, *Borrelia* spp., *Rickettsia* spp., *Anaplasma phagocytophilum* and *Coxiella burnetii* are the most important in northern Spain due to their clinical and epidemiological interest [6,7].

Lyme borreliosis (LB), a zoonotic disease caused by *B. burgdorferi* sensu lato (s.l.), is the most common TBP on humans and animals in Europe [8]. While the overall prevalence of LB may be stabilizing, its geographical distribution continues to expand [9]. In Europe, five genospecies of *B. burgdorferi* s.l. (*B. afzelii*, *B. garinii*, *B. burgdorferi* sensu stricto (s.s.), *B. spielmanii* and *B. bavariensis*) can cause the disease in humans [10,11,12]. In addition, other pathogenic genospecies such as *B. bissettii*, *B. lusitaniae* and *B. valaisiana* have sporadically been detected in patients [13,14,15], but are not recognized as important pathogens. In some areas of the Basque Country, LB is endemic [16] and five genospecies (*B. afzelii*, *B. garinii*, *B. burgdorferi* s.s., *B. lusitaniae* and *B. valaisiana*) have been identified in its main vector, *Ixodes ricinus* [16,17]. This tick species is also the vector of *Borrelia miyamotoi*, a zoonotic member of the relapsing fever group of TBPs [18]. Despite the increasing detection of *B. miyamotoi* across the Northern Hemisphere, its incidence in humans remains poorly studied, with most cases presenting mildly but potentially leading to severe disease in immunocompromised individuals [19]. Although this species has not yet been reported in the Basque Country, it has been detected in neighbouring regions such as La Rioja [20], suggesting the need to consider it in surveillance studies.

Rickettsioses are the oldest known infectious diseases [21]. In Europe, the majority of rickettsial infections are caused by tick-transmitted rickettsiae belonging to the spotted fever group (SFG) [6,7]. This group includes *Rickettsia conorii* subsp. *conorii*, the causative agent of Mediterranean spotted fever (MSF), as well as other *Rickettsia* spp. that cause MSF-like illnesses, such as *R. helvetica*, *R. monacensis*, *R. massiliae* or *R. aeschlimannii* [7,22]. Some years ago, *R. monacensis* was identified as the causative agent of illness in one patient from the Basque Country [23]. Additionally, several discovered rickettsiae of unknown pathogenicity have been reported in ixodid ticks removed from humans in Spain [24,25,26,27,28]. To date, only *R. raoultii* has been detected in ixodid questing ticks in the Basque Country [29], although in other regions of northern Spain, several rickettsial species (“*Candidatus* Rickettsia rioja”, *R. raoultii* and *R. slovaca*) have been detected in ticks [7,30,31].

*Anaplasma phagocytophilum*, a zoonotic bacterium, causes human granulocytic anaplasmosis (HGA), tick-borne fever in domestic ruminants, equine granulocytic anaplasmosis in horses and granulocytic anaplasmosis in dogs and cats [32,33]. The infection has been detected in several mammalian species, including humans, in areas on the Northern Hemisphere where *I. ricinus* is endemic and considered the primary vector [34,35]. In the Basque Country, *A. phagocytophilum* has been detected in questing ticks [29] and in domestic and wild animals [36,37,38]. Although several genetic variants of *A. phagocytophilum* with varying pathogenicity and host association have been identified [33], their presence and distribution remain largely uncharacterized in the Basque Country.

*Coxiella burnetii* is particularly relevant in northern Spain, where Q Fever pneumonia is endemic [39] and large outbreaks have been identified [40,41]. Over 40 tick species are found to be naturally infected with *C. burnetii*, suggesting that they may play a role in its transmission to vertebrate hosts in nature [42,43]. In the Basque Country, however, ticks do not appear to play a major role in the cycle of the disease [29].

Although several studies in the Basque Country have documented the diversity, seasonal dynamics and zoonotic TBPs in questing ticks [5,17,29] and those collected from non-human animals [16,36,44], no research to date has specifically addressed ticks biting humans. Existing on-host investigations have focused on livestock and wildlife, providing valuable ecological and epidemiological insights, but leaving a critical gap regarding human exposure. Understanding which tick species are feeding on humans, and when and where their bites occur, is essential to accurately assess the risk of TBD and to guide public health interventions in the region. The objective of this study is the identification of tick species biting humans and the detection of tick-borne zoonotic bacteria through the molecular analysis of specimens collected from individuals who attended hospitals and health centres of the Basque Health Service–Osakidetza primarily for tick removal.

## 2. Materials and Methods

### 2.1. Tick Collection and Identification

Between 2019 and 2024, ticks removed from humans have been collected throughout the year in hospitals and health centres across the Basque Country, northern Iberian Peninsula. These arthropods were identified at the species level based on morphological characteristics as soon as they were received [45], using an SMZ 18 stereo microscope (Nikon, Amstelveen, The Netherlands), and classified according to their life development stage (adult, nymph or larva) and feeding status (unfed or fed). Ticks that could not be confidently identified to the species level using morphological criteria were subjected to cytochrome oxidase I (COI) gene sequencing (406 bp amplicon) (Macrogen, Seoul, Republic of Korea) and homology-based identification using BLAST (https://blast.ncbi.nlm.nih.gov/Blast.cgi, accessed on 22 January 2025), during the final year of the study. Ticks submitted by the health centres were stored at −20 °C until molecular analyses were performed. For the pathogen detection analyses, adult ticks were analysed individually, while nymphs and larvae were pooled—up to two nymphs or three larvae per pool—if provided they were collected from the same patient.

### 2.2. DNA Extraction

DNA extraction was carried out on collected specimens in pools of one, two or three individuals, grouped by patients when multiple ticks were collected from the same individual, as well as by species and life stage. Tick pools were homogenized in a 1.5 mL screw-cap vial with 400 µL of TE buffer (10 mM Tris, EDTA 1 mM pH = 8) in a TissueLyser II (Qiagen, Hilden, Germany) at 30 Hz for 10 min using 2.3 mm steel beads. The extraction was performed using 200 µL of the tick homogenate, 100 µL of 10% Chelex ^®^ 100 sodium form (Sigma-Aldrich, St. Louis, MO, USA) resin and 0.15 mg of Proteinase K lyophilized (PanReac AppliChem, Darmstadt, Germany). The mixture was incubated at 56 °C for 30 min on a heating block, vortexed and then incubated at 95 °C in a thermostatic water bath for 10 min.

After centrifugation at 13,300 rpm for 10 min, the supernatant was transferred to a sterile 1.5 mL Eppendorf vial. A volume of 100–150 µL was recovered, avoiding carryover of 10% Chelex ^®^ 100 sodium form beads. If additional volume was required, a second centrifugation under the same conditions was performed. The extracted DNA was stored at −40 °C until further processing. A negative control consisting of TE buffer was used for every 10 samples to verify that there was no contaminating DNA during the DNA extraction process.

To rule out the presence of PCR inhibitors in DNA samples, a conventional PCR of the COI gene [46] was used as internal control.

### 2.3. Molecular Detection and Identification of Tick-Borne Bacteria

The extracted DNA was subsequently analysed using various real-time PCRs to detect the presence of *Borrelia* spp., *Rickettsia* spp., *A. phagocytophilum* and *C. burnetii*.

A real-time duplex PCR was adapted for the specific detection of the *Borrelia* spp. and *Rickettsia* spp. using the 5S-23S intergenic spacer (IGS) and the *glt*A gene as target sequences, respectively, based on previously designed primers and probes [45,46]. For *Borrelia* spp., IGS-MGB *Borrelia* forward 5′-TCCTAGGCATTCACCATAGACT-3′, IGS-MGB *Borrelia* reverse 5′-TGGCAAAATAGAGATGGAAGAT-3′, IGS-MGB *Borrelia* probe 5′-[FAM]ATTACTTTGACCATATTT[MGBEQ]-3′ [47]. Regarding *Rickettsia* spp., CS-Forward 5′-TCGCAAATGTTCACGGTACTTT-3′, CS-Reverse 5′-TCGTGCATTTCTTTCCATTGTG-3′, CS-Probe 5′-[CY5]TGCAATAGCAAGAACCGTAGGCTGGATG[BHQ2]-3′ [48]. Although the real-time PCR assay was originally designed to detect *B. burgdorferi* s.l., it has also been shown to detect *B. miyamotoi* [49]. Therefore, positive samples were further analysed using species-specific methods to accurately identify the *Borrelia* species present, as described below. The *Borrelia* spp. probe (FAM-MGBEQ) and *Rickettsia* spp. probe (Cy5-BHQ2) have their fluorophores attached at the 5′ end and quenchers at the 3′ end, respectively. The PCR reaction was carried out using Premix Ex TaqTM (Takara Bio, Kusatsu, Shiga, Japan), 400 nM of each primer and 200 nM of each probe and 20 mg/mL of BSA for each reaction, and 3 µL of extracted DNA to a total reaction volume of 15 µL. The reactions were performed and analysed using an Applied Biosystems QuantStudio 5 Real-Time PCR System (Applied Biosystems™, Life Technologies, Carlsbad, CA, USA), with an initial temperature of 95 °C for 30 s, followed by 40 cycles of 95 °C for 15 s of annealing and 60 °C for 1 minute of extension. For the specific detection of the *B. burgdorferi* genospecies, a reverse line blot (RLB) was performed [50]. Positive DNA samples were analysed by a PCR targeting the 5S-23S rRNA IGS (PCR 5S-23S) followed by RLB with eight species-specific probes for *B. burgdorferi* s.l., *B. burgdorferi* s.s., *B. afzelii*, *B. garinii*, *B. bissettii*, *B. spielmanii*, *B. valaisiana* and *B. lusitaniae* [16,50,51,52]. Some samples positive for *B. burgdorferi* s.l. by real-time duplex PCR but negative by 5S-23S PCR were subsequently subjected to generic 16S rRNA PCR and sent for Sanger sequencing (Macrogen, Seoul, Republic of Korea). To identify the *Rickettsia* species in the positive samples, the *glt*A and *omp*A genes were amplified by conventional nested PCR [53,54,55], and subsequently sequenced by Sanger methods (Macrogen, Seoul, Republic of Korea).

In order to detect the presence of *A. phagocytophilum* and *C. burnetii*, previously described real-time PCR protocols were carried out, targeting the *msp2* [56,57] and *IS1111* genes, respectively [58]. Although the IS1111 protocol was originally designed to amplify *C. burnetii*, it is known to also detect *Coxiella*-like endosymbionts [59] and therefore results were reported conservatively as *Coxiella* spp.

To avoid cross-contamination, DNA extraction, mixing of DNA-free PCR reagents and template DNA addition were performed in separate areas with separate equipment and solutions. For PCR and RLB protocols, ultrapure water (Sigma-Aldrich, St. Louis, MO, USA) was used as negative PCR control and specifically designed plasmids for each bacterium were used as positive controls (Eurofins Genomics, Ebersberg, Germany).

Sequences obtained in this study were then compared to those in GenBank using BLAST analysis (https://blast.ncbi.nlm.nih.gov/Blast.cgi, accessed on 22 January 2025). The consensus sequence for PCR products was generated using BioEdit v7.1.3 software.

## 3. Results

### 3.1. Tick Species, Stage and Season

A total of 181 ticks were collected from 148 patients, including 6 larvae (3.3%), 128 nymphs (70.7%) and 47 adults (26%), of which 40 (22.1%) were females and 7 (3.9%) were males (Table 1). The collected ticks belonged to four different genera, *Ixodes* (161, 89.0%), *Dermacentor* (8, 4.4%), *Rhipicephalus* (6, 3.3%) and *Haemaphysalis* (6, 3.3%). Nymphs belonged exclusively to the genera *Ixodes* and *Dermacentor*, and larvae only to *Ixodes*.

Among the *Ixodes* specimens, all were identified as *I. ricinus.* Two morphologically unidentifiable females, due to missing appendages or damaged scuta, were confirmed as *I. ricinus* via COI gene sequencing (100% identity). Two species of *Dermacentor* were identified: *Dermacentor reticulatus* (2.8%) and *Dermacentor marginatus* (1.7%). *Rhipicephalus bursa* (3.3%) and *Haemaphysalis punctata* (3.3%) were the only species identified from their respective genera.

The adult stage was the most frequently collected across all genera (for non-*Ixodes*: 90.5% adults and 9.5% nymphs), except for *Ixodes*, in which nymphs accounted for most specimens (26.4% adults, 78.3% nymphs and 3.7% larvae). All adults of *I. ricinus* were females, while in the other species both females and males were collected. Ticks were collected throughout the year, with the highest abundance recorded in spring (38.6%) and summer (49.7%), particularly in June and July, when 91.1% of all summer-collected ticks were recovered. Of all collected ticks, 63 (35%) showed some evidence of feeding, with partial or full increase in body size indicating blood ingestion, and attachment to a host.

### 3.2. Pathogen Detection and Identification

In total, 22 pools of two ticks and one pool of three ticks were analysed, along with 134 individual specimens, resulting in 157 pathogen detection analyses. All the ticks analysed for the COI gene, used as an internal control to assess DNA quality and the absence of PCR inhibitors, showed a positive result.

DNA from the studied pathogens was detected in 25 samples or pools (15.9%), all consisting of a single specimen except for two pools, each of which contained two *I. ricinus* nymphs. Of these, ten (6.4%) were positive for *Borrelia* spp., eight (5.1%) for *Rickettsia* spp., four (2.5%) for *A. phagocytophilum* and three (1.9%) for *Coxiella* spp. (Table 1).

Among *Borrelia* spp. positive samples, seven (8.7% of the *I. ricinus* nymphs analysed) hybridised with probes targeting the *B. burgdorferi* s.l. complex. Regarding the *B. burgdorferi* genospecies detected in the RLB, *B. afzelii* was identified four times, *B. garinii* and *B. valaisiana* twice and *B. burgdorferi* s.s. once (Table 2). Mixed *Borrelia* infections were found in two samples: one containing a single nymph testing positive for *B. afzelii* and *B. burgdorferi* s.s., confirming a true coinfection; and another consisting of a pool of two nymphs testing positive for *B. afzelii* and *B. garinii*, in which coinfection at the individual level could not be confirmed. Furthermore, two samples of *I. ricinus* nymphs positive in the *Borrelia-Rickettsia* real-time duplex PCR but negative in the RLB were identified as *B. miyamotoi* by Sanger sequencing of the 16S gene. These sequences showed 100% identity with a *B. miyamotoi* sequence previously detected in *I. ricinus* from Austria (GenBank Accession Number: PV203587.1) and represent 1.9% of the *I. ricinus* nymphs analysed in this study. In contrast, the sequence obtained from a male of *H. punctata* showed 100% identity with a *Borrelia* sp. sequence previously detected in a *H. punctata* from France (Genbank Accession Number: MW301926) [60].

Among *Rickettsia* spp., *R. monacensis* was identified in three samples of *I. ricinus* nymphs and *R. helvetica* in another. *“Candidatus* R. rioja” was detected in two adults of *D. reticulatus* and in one nymph of *D. marginatus* and *R. massiliae* was identified in one adult of *R. bursa* (Table 3).

Coinfection with two pathogen species was found in one *I. ricinus* nymph, which carried a combination of *A. phagocytophilum* and *R. helvetica*. A mixed infection with three species (*R. monacensis*, *B. burgdorferi* s.s. and *B. afzelii)* was detected in another *I. ricinus* nymph.

Pathogen detection showed variation across seasons in the percentage of positive samples, but this should be considered in the context of the lower number of samples analysed in autumn and winter (Table 1). Detection of at least one pathogen was recorded in every season, with *Borrelia* spp., being the only pathogen present throughout the entire year.

## 4. Discussion

The emergence and re-emergence of TBD in recent years highlights the need to understand tick population distribution and the detection of TBPs. The presence of four groups of human tick-borne bacteria was investigated in ixodid ticks from Basque Country (northern Spain) between 2019 and 2024 using molecular methods.

The predominance of *I. ricinus* among ticks collected from patients attending healthcare centres is consistent with previous vegetation and host-based tick surveys conducted in northern Spain [5,36,37,61,62]. The high occurrence of *I. ricinus* aligns with the humid and mild climate typical of the Basque region, which favours the survival and development of this tick species [29]. Furthermore, the three stages of development of this species are exophilic, therefore each development stage presents a potential risk for human exposure. However, although the larvae represent the most abundant stage [5,61,63,64], in our study the nymph stage was the most frequently observed in humans.

The number of specimens from other species was small compared to *I. ricinus*. Although the exact region of exposure in unknown, *R. bursa* deserves to be highlighted, as it is a more xerophilic species and typically less abundant in this region. Despite this, a similar number of specimens were collected as for *H. punctata* or *D. reticulatus*, which are more hydrophilic and generally more abundant in the area [61]. In the study carried out by Vieira et al. [65] in the neighbouring region of Castilla y León, *R. bursa* was the second most abundant species collected from patients. These findings suggest that this species may have a notable affinity for feeding on humans.

*Ixodes ricinus* has a wide host range at all stages of development [66]. Larvae usually feed on small vertebrates [67], nymphs on small and large vertebrates [68], while adults frequently feed on larger mammals [69]. These feeding habits, together with the high abundance of these tick species in this region, may be the reason why nymphs are the most frequently collected stage, rather than larvae.

The duration of tick attachment is crucial for pathogen transmission and risk assessment of TBD [70]. In our study, around one-third of the ticks were classified as fed, indicating that the majority were removed before initiating blood feeding and therefore posed a limited risk to patients. In the Basque Country there is a strong tradition of outdoor activities in the mountains such as hiking, mushroom foraging, hunting or forestry work, which increases the likelihood of tick exposure. However, self-checks for ticks upon returning home are becoming more common among the population engaged in outdoor activities. This may explain the reason why most of the ticks removed from patients were unfed [70].

In this geographical region, ixodid ticks are active all year round [5] and since leisure activities are also carried out throughout the year, ticks can be collected in any month. However, the number of ticks removed was higher in spring and summer than in autumn and winter. These results are consistent with those reported in a neighbouring region [65], and in addition to the fact that outdoor activity may be higher during these seasons.

In addition to being the most abundant tick species in northern Spain and Europe, *I. ricinus* is recognised as a major vector, transmitting over twenty different potentially pathogenic parasites, bacteria and viruses to vertebrate hosts. The zoonotic multisystemic illness known as LB is considered the most prevalent TBD worldwide [8] and its primary vector is *I. ricinus*. In northern Spain, there are endemic areas of LB, coinciding with the distribution of this Ixodidae tick, in which five genospecies have been identified: *B. burgdorferi* s.s., *B. garinii*, *B. afzelii*, *B. valaisiana* and *B. lusitaniae* [16]. In line with this pattern, the first four genospecies were found in *I. ricinus* nymphs, while no detection of *B. lusitaniae* was observed. Of particular interest, two *B. miyamotoi*-positive ticks were detected after removal from patients who sought healthcare in the Basque Country, where no prior detections of this pathogen had been reported, although the exact place of exposure could not be determined. The observed prevalence was three to four times higher than that reported in other regions of northern Spain [20,71,72]. In contrast to these previous studies, none of the *I. ricinus* females were positive for *Borrelia* spp., although in this case the small number of available samples may have limited the detection. However, the *Borrelia* prevalence detected in nymphs (8.7%) was higher than that reported in previous studies in questing ticks in the Basque Country (1.5–2%) [16,17] and that found in La Rioja (4.6%) [20], similar to that found in Galicia (10%), northwestern Spain [71], but lower than that reported in more northern European countries [73]. The higher prevalence currently detected suggests that the risk of *B. burgdorferi* s.l. transmission may have increased in recent decades in the Basque Country.

Herein, we also report that *Rickettsia* was the second most commonly identified group of bacteria, detected across multiple tick species. The most prevalent SFG *Rickettsia* species was *R. monacensis*, consistently reported across Europe together with its main vector, *I. ricinus* [74]. The prevalence of infected ticks in Spain reaches 6–27% [31,75]. Four cases have been reported in Europe so far [7], but it is interesting to note that one of the two cases diagnosed in Spain have been in the Basque Country [23]. In addition, *R. helvetica*, a human-pathogenic SFG *Rickettsia* previously reported in other parts of northers Spain [24], was detected in *I. ricinus* nymphs. This represents the first detection of this pathogen in a tick retrieved from a person who sought healthcare in the Basque Country. [24]. However, unlike in other European regions, it has not yet been reported in humans in the Iberian Peninsula, to the best of our knowledge [76]. Another SFG-*Rickettsia* identified in this study was *R. massiliae*, a species typically associated with ticks of the *R. sanguineus* complex [7], although in our study it was detected in a female *R. bursa*. The detection of one positive among only six specimens analysed suggests a high prevalence of this SFG *Rickettsia* in *R. bursa.* Nevertheless, a larger sample size would be required to confirm this observation. Furthermore, the presence of “*Candidatus* R. rioja” was also identified on three occasions. This agent is one of the involved in the group of diseases known as TIBOLA/DEBONEL/SENLAT, whose main vectors are ticks of the genus *Dermacentor*, mainly *D. marginatus* [7]. However, in northern Spain it has also been found in other genera [30,31]. In our case, they were found in two adult specimens of *D. reticulatus* and in a nymph of *D. marginatus*.

*Ixodes ricinus*, the primary vector of *A. phagocytophilum* in Europe [6], also tested positive in the present study, with the prevalence slightly lower than that previously reported in the Basque Country [29]. Unlike in the United States, very few cases of HGA have been reported in Spain and elsewhere in Europe, which may reflect the lower pathogenicity of the circulating strains, vector differences (*I. ricinus* vs. *Ixodes scapularis*) [77] and, in the case of Spain, generally low prevalence rates in ticks [29,30,71].

Although ticks are suggested both reservoir and vector of *C. burnetii* [42,43], with higher infection rates reports in Central Spain in ticks collected from animals and questing ticks [78], available data from the Basque Country suggest a low circulation of the pathogen in local tick populations. Low infection rates in questing ticks collected from vegetation, as well as the absence of *C. burnetii* in adult ticks removed from wild animals, have been previously reported in the Basque Country [29] and in another region of northern Spain [30]. These findings align with our results, suggesting that ticks do not appear to play a significant role in the transmission cycle of *C. burnetii* in this area. It should be noted that the PCR protocol used may also amplify *Coxiella*-like endosymbionts, which are genetically similar but non-pathogenic. If the three ticks testing positive for *Coxiella* were indeed carrying such endosymbionts instead of *C. burnetii*, this would further support the hypothesis that ticks play a negligible role in the local epidemiology of this pathogen.

Co-occurrences of different pathogens in *I. ricinus*, highlights the possibility of simultaneous infections. In the study, examples include *R. monacensis*, *B. burgdorferi* s.s. and *B. afzelii*, representing a triple infection, as well as dual infection with *R*. *helvetica* and *A. phagocytophilum*. The combination of *R. monacensis* and *B. burgdorferi* s.l. had already been reported in questing ticks in a previous study [30]. These coinfections may result from the co-transmission of pathogens during blood feeding on the host [79] or from infections acquired across multiple feedings. The presence of multiple pathogens highlights a potential risk for both humans and animals to contract coinfections, which should be considered in the diagnosis and treatment of TBD. It should be noted that no personal information was available for the patients included in this study (e.g., travel history or animal contact), so it is possible that some of the ticks were acquired outside the Basque Country. Nevertheless, the presence of these pathogens remains relevant for regional public health services, as any potential disease would likely manifest and require diagnosis and treatment within the region.

## 5. Conclusions

This study provides valuable insight into the growing body of evidence on the presence of the tick species parasitizing humans in the Basque Country, the diversity of zoonotic pathogens they may carry and the circulation of TBPs in northern Spain. While the exact region of tick acquisition cannot be confirmed, the first detection of *B. miyamotoi* and *R. helvetica* in ticks removed from individuals seeking healthcare in the Basque Country supports the value of patient-derived ticks as a tool for early detection and surveillance of emerging TBPs relevant to regional public health. To improve the interpretation of entomological data in future surveillance efforts, incorporating a standardized epidemiological questionnaire is recommended, as it would provide valuable context on potential exposure pathways. In addition, the detection of coinfections, including cases involving three zoonotic bacteria, reflects the complexity of TBP circulation. Altogether, this work underlines the value of integrating molecular diagnostics with passive surveillance based on human-attached ticks as a practical and informative tool for regional tick and pathogen monitoring within a One Health framework. Future research should focus on factors influencing human exposure to ticks and TBPs to optimize surveillance efforts and better inform public health strategies.

## Figures and Tables

**Table 1:** Number of tick samples by species and developmental stage and season of collection, along with results of pathogen detection, including the number of positive samples and, in parentheses, the percentage of positivity relative to the total number of tick samples analysed for each species, stage and/or season.

	Analysed Samples	*A. phagocytophilum*	*Coxiella* spp.	*Borrelia* spp.	*Rickettsia* spp.
Tick species/stage					
*I. ricinus*	137	4 (2.9)	3 (2.2)	9 (6.6)	4 (2.9)
Female	29	1 (3.4)	0	0	0
Nymph	104 *	3 (2.9)	3 (2.9)	9 (8.7)	4 (3.8)
Larvae	4 *	0	0	0	0
*R. bursa*	6	0	0	0	1 (16.7)
Female	3	0	0	0	1 (33.3)
Male	3	0	0	0	0
*H. punctata*	6	0	0	1 (16.7)	0
Female	4	0	0	0	0
Male	2	0	0	1 (50.0)	0
*D. reticulatus*	5	0	0	0	2 (40.0)
Female	3	0	0	0	1 (33.3)
Male	2	0	0	0	1 (50.0)
*D. marginatus*	3	0	0	0	1 (33.3)
Female	1	0	0	0	0
Nymph	2	0	0	0	1 (50.0)
Season					
Spring	63	2 (3.2)	2 (3.2)	5 (7.9)	5 (7.9)
Summer	73	2 (2.7)	1 (1.4)	3 (4.1)	2 (2.7)
Autumn	10	0	0	1 (10.0)	0
Winter	11	0	0	1 (9.1)	1 (9.1)
Overall	157	4 (2.5)	3 (1.9)	10 (6.4)	8 (5.1)

* Note that one *I. ricinus* larva sample corresponds to a pool of three larvae and that twenty-two of the *I. ricinus* nymph samples correspond to pools of two nymphs each.

**Table 2:** Identification of *Borrelia* species based on RLB and 16S gene sequences obtained in this study.

Tick Species-S	No.	RLB	Sanger Sequencing
*I. ricinus*-N	1	*B. afzelii*	-
*I. ricinus*-N	1	*B. afzelii*	-
*I. ricinus*-N	1	*B. afzelii* + *B. burgdorferi* s.s.	-
*I. ricinus*-N	2	*B. afzelii* + *B. garinii*	-
*I. ricinus*-N	1	*B. garinii*	-
*I. ricinus*-N	1	*B. valaisiana*	-
*I. ricinus*-N	2	*B. valaisiana*	-
*I. ricinus*-N	1	neg	*B. miyamotoi*
*I. ricinus*-N	1	neg	*B. miyamotoi*
*H. punctata*-M	1	neg	*Borrelia* sp.

S, stage: M, male or N, nymph; No., number of tick specimens in the analysed sample; neg, negative.

**Table 3:** Identification of *Rickettsia* species based on *glt*A and *omp*A gene sequences obtained in this study. For each positive sample, the amplified gene, sequence length, closest match in GenBank and the consensus species identification are shown. All sequences showed 100% query coverage and identity with the corresponding GenBank sequences. All samples corresponded to a single tick specimen.

Tick Species-S	*glt*A			*omp*A			Consensus
	Pb	AN	Species	Pb	AN	Specie	
*I. ricinus*-N	309	MK792589	*R. monacensis*	421	KY073124	*R. monacensis*	*R. monacensis*
*I. ricinus*-N	311	OR399958	*R. monacensis*	494	HM161767	*R. monacensis*	*R. monacensis*
*I. ricinus*-N	311	MK792589	*R. monacensis*	nrs	-	*-*	*R. monacensis*
*I. ricinus*-N	312	MK792578	*R. helvetica*	nrs	-	*-*	*R. helvetica*
*R. bursa*-F	304	PP835452	*R. massiliae*	490	MZ420708	*R. massiliae*	*R. massiliae*
*D. marginatus*-N	296	AY129300	*Rickettsia* sp.	489	MW817120	*Ca* R. rioja	*Ca* R. rioja
*D. reticulatus*-F	nrs	-	-	372	MW817127	*Ca* R. rioja	*Ca* R. rioja
*D. reticulatus*-M	nrs	-	-	423	MW817127	*Ca* R. rioja	*Ca* R. rioja

S, stage: F, female, M, male or N, nymph; Pb, pair base size; AN, accession number; nrs, non-readable sequences.

## Data Availability

The data presented in this study are available within the article.

## Abbreviations

The following abbreviations are used in this manuscript:

COI	Cytochrome c oxidase subunit I
DNA	Deoxyribonucleic acid
*glt*A	Citrate synthase gene
HGA	Human granulocytic anaplasmosis
IGS	Intergenic spacer
*IS1111*	IS1111 gene
LB	Lyme borreliosis
MSF	Mediterranean spotted fever
*msp2*	Merozoite surface protein 2
*omp*A	Outer membrane protein A gene
PCR	Polymerase chain reaction
RLB	Reverse line blot
s.l.	sensu lato
s.s.	sensu stricto
SFG	Spotted fever group
TBD	Tick-borne disease
TBP	Tick-borne pathogen
